# Sustainable Development Evaluation on the Landscape Design of Industrial Heritage Park: A Case Study of Tao Sichuan, China

**DOI:** 10.1155/2023/1564614

**Published:** 2023-07-07

**Authors:** Lei Meng, Bao-gang Lin, Hui-zhong Zhang, Rui Bu

**Affiliations:** ^1^School of Art, Xi'an University of Architecture and Technology, 710000 Xi'an, China; ^2^School of Mechanical Engineering, Northwestern Polytechnical University, 710000 Xi'an, China

## Abstract

The sustainable development of the urban environmental landscape is a process that integrates resource utilization, ecological benefit, economy, and society and involves elements of culture, society, politics, economy, and individual residents. Citizen participation is increasingly important for the urban landscape design, and therefore, urban environmental landscape studies must be evaluated objectively by the public to ensure both sustainable development and social justice. In this study, the Tao Sichuan Creative Industry Park (the former “Universe Porcelain Factory”) in Jingdezhen, Jiangxi province, China, was taken as example, and the implemented landscape reconstruction was evaluated. The analytical hierarchy process was used to collect both expert and public opinions regarding the cultural landscape; then, the weight coefficient of the value index layer of the industrial heritage park was obtained. A comparison of the two groups (experts and the public) showed that the experts do not exactly agree with the perspective of the public: experts prefer the artistic value far more than any other factors, while the public prefers the artistic value, social value, and economic value. Each group prefers different values of the landscape, suggesting that environmental justice should not be biased toward one of these perspectives. Finally, a design optimization principle is proposed according to the results of this study. This principle strengthens the sustainable development concept in the landscape reconstruction of industrial heritage parks, and suggestions are provided for optimizing the allocation of urban public landscape resources.

## 1. Introduction

The definition, value, legal protection, and conservation of industrial heritage have been elaborated in the Charter of Nizhny Tagil [1]. In 2010, the China Urban Planning Society held a seminar on the protection and reuse of urban industrial heritage, jointly emphasizing industrial heritage protection, economic development, and urban regeneration [2]. The roles of industrial facilities, industrial sites, and industrial landscapes have changed over time, from carriers of industrial production to abandoned sites and relics. To avoid the disappearance of China's rich and distinctive modern industrial heritage resources in today's large-scale urban renewal movement, research identifying the “heritage” value of industrial heritage is booming [3]. As of 2016, a total of 329 industrial heritage sites in China have been incorporated into the “Chinese Industrial Heritage Protection List” and are now effectively protected by law. However, this number represents only 7.66% of the total number of cultural heritage units, reflecting the public's vague perception of the value of “cultural relics” of industrial heritage [4]. However, a large number of the remaining cultural heritage units that have not been included in the “cultural insurance unit” are still facing severe survival threats, and a protection basis beyond the identity of “cultural insurance” is urgently needed. Therefore, research is needed on how to overcome the limitations of the historical authenticity perspective. The “nonmemory” and “practical” characteristics of industrial heritage, which differ from “cultural relics”, need to be combined to explore a new sustainable development mode for industrial heritage sites. Such an exploration is urgently needed in the field of industrial heritage protection.

In 2018, the Ministry of Industry and Information Technology released the first batch of a total of 100 national industrial heritage sites, including the state-run “Universe Porcelain Factory” in Jingdezhen, Jiangxi province, China. Jingdezhen, which has been the capital of porcelain production for thousands of years, became the center of industrial ceramic production after the founding of New China. As one of the top 10 state-owned porcelain factories, the industrial elements of this heritage site reflect the history of the city and the feelings and pride of its residents. However, existing research on the construction development and design of the industrial heritage landscape in Jingdezhen has not focused on sustainable development and the landscape. To fill this gap, in this study, a cultural landscape assessment hierarchy is constructed based on the principle that the existing cultural landscape of the park reflects its heritage value. Combined with the results of the research on the evaluation of the heritage value of the park by all people and their feelings, the opinion of landscape improvement is formed based on the principle of meeting the sustainable development of the landscape.

### 1.1. Sustainable Development of the Urban Landscape

The concept of “sustainable development” comprises two meanings: “Sustainability” means maintaining the objective regularity and possibility for the sustainable development of nature and human society to avoid artificial interruption [5]. To ensure that contemporary and future generations have the same usage right, it is necessary to protect existing material and cultural resources. “Development” means seeking a suitable development path based on ensuring “sustainability” so that human society can develop in a more civilized and progressive way. Therefore, “sustainable development of the urban landscape” can be understood as the preservation of valuable traditional urban landscapes to ensure their continuity and development into the future. At the same time, a good urban landscape environment should be created to promote the orderly and healthy development of urban landscapes and meet the continuous physical and psychological development needs of urban residents. [4] emphasized the “need” when proposing the concept of urban landscape to help implement intervention measures and achieve greater social justice. In addition, landscape research based on demand also promotes the sustainable development of society. In a study of the cultural landscape of the Röhn Biosphere Reserve in Germany, Büttner and Dongyi proposed that via the cognition and analysis of the cultural landscape, people can reach a consensus on the comprehensive regional characteristics of history, society, and aesthetics [6]. Zhang and He found that with the emergence of environmental impacts as well as both social and health consequences, environmental challenges in cities have induced explorations of feasible strategies for building resilient, sustainable, healthy, and livable built environments [7, 8]. Based on this consensus, sustainability strategies, research projects, and economic development that protect and nurture cultural landscapes can be implemented effectively. Liu et al. proposed that urban landscape design should be based on the landscape ecology theory and sustainable development and should focus on the combination of landscape dynamic patterns with nature, science, and art [9].

### 1.2. The Cultural Landscape of Industrial Heritage

As one among many landscape types, the industrial landscape reflects the relationship between people and machines, as well as between architecture and nature. It embodies industrial culture and industrial civilization, including the overall environment composed of industrial equipment and industrial facilities that demonstrate both industrial technology and processes [10]. Li Jianbin has divided the industrial landscape into an old industrial landscape and a new industrial landscape. The old industrial landscape refers to the industrial heritage landscape [11], which can be modified and reused [12], while the new industrial landscape refers to the “postindustrial landscape” [13]. This postindustrial landscape is a new landscape that is redesigned and built on industrial relics or abandoned sites.

In recent years, research on the overall protection of industrial heritage from a landscape perspective has gradually emerged in China. Related concepts include the “industrial heritage corridor” and “industrial heritage area” [14–16]. The criteria for heritage identification are actually a series of values. With the transformation of the value cognition of industrial heritage from “cultural relics” to “landscape,” the value composition, ranking, and proportion of industrial heritage also change. Research on industrial heritage cultural landscapes mainly focuses on the construction of the landscape space based on functional orientation or on landscape restoration technology based on environmental engineering. From the perspective of landscape, the identity of heritage is determined not only by the inherent value of the heritage itself but also by the selection and management of the external environment [17]. This precisely reflects the importance of the “systematic value” generated by incorporating the individual value into the concept of overall sustainable development. Shufang et al. constructed landscape design methods based on the morphological identification and module division of industrial waste [18]. Regarding the study of the cultural landscape of the industrial heritage of Tao Sichuan, Yuan proposed to incorporate the important role of the landscape design in the symbolic meaning of the cultural space in research on the mechanism of the recreation of the cultural space in Tao Sichuan [19]. Wang proposed landscape production in the urban art district of Tao Sichuan [20].

Landscape justice has become an important aspect of sustainable environmental development [21]. However, existing research on landscape justice of the industrial heritage value is limited, and available studies mainly focused on the design and practice of a certain landscape element. In addition, existing research basically starts from the perspective of landscape architecture decision makers and lacks research and design practice based on the views of the audience. Based on landscape decision makers and the public's perception of the cultural landscape of Tao Sichuan, this study used the analytic hierarchy process (AHP) to analyze and identify the needs of cultural landscapes in industrial heritage value expression and social justice. Perspectives and methods are provided for the landscape design of industrial heritage parks (especially light industry heritage parks).

### 1.3. Status of Tao Sichuan Creative Industry Park

**Figure 1:**
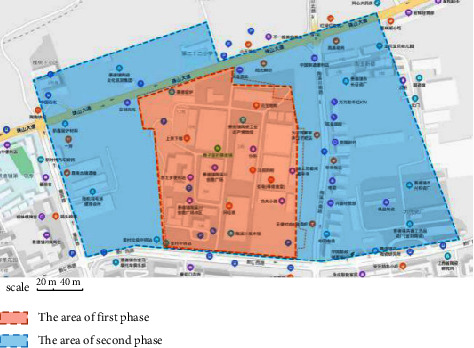
Planning map of the Tao Sichuan Creative Industry Park.

**Figure 2:**
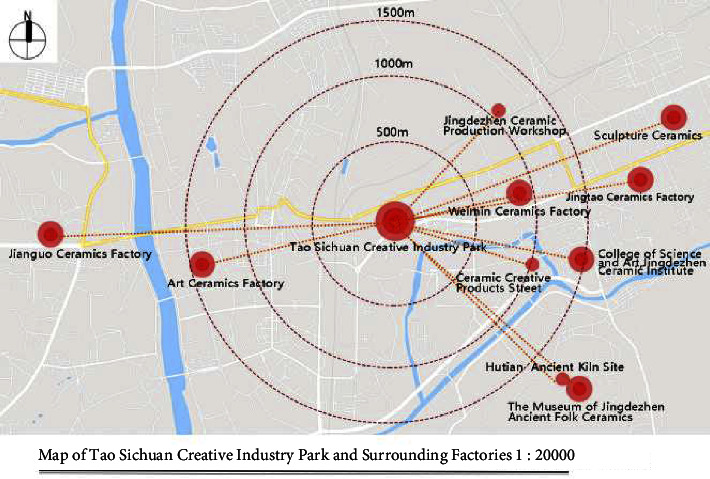
Geographical location of the Tao Sichuan Creative Industrial Park in Jingdezhen city.

**Figure 3:**
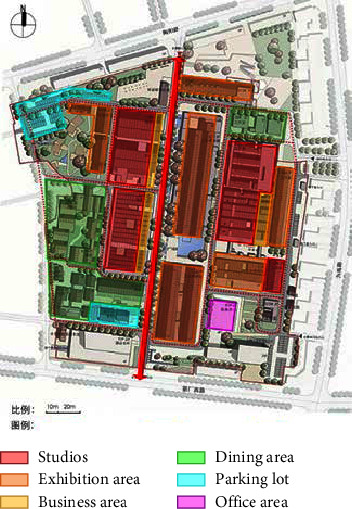
Map of the functional distribution of Tao Sichuan Creative Industrial Park.

As a ceramic production area, Jingdezhen has a history of thousands of years, and since the Han Dynasty, it has been the land of official kilns. In 1949, New China provided investments to Jingdezhen to build a batch of state-owned porcelain factories, one of which was the Universe Porcelain Factory. From the 1950s to the 1980s, the Universe Porcelain Factory had 22 ceramic industrial plants and a complete ceramic production process chain. This chain consisted of ceramic molding lines, waste heat utilization systems, industrial pipes, heating pipe supports, and other industrial equipment and facilities as well as production material resources. Because of the reform of state-owned enterprises in the 1990s, Tao Sichuan was basically abandoned after 1996. The Universe Porcelain Factory has made important contributions to the construction and development of China's ceramic industry and has shaped the landscape of the industrial heritage. In 2012, Jingdezhen took the transformation of the old industrial zone as driver of urban rejuvenation efforts [22] and the Universe Porcelain Factory, which was renamed to “Tao Sichuan Creative Industry Park,” became a key pilot project (Figures 1 and 2). At present, the Tao Sichuan phase I project covers an area of more than 270 mu, with a total construction area of 180,000 m^2^ (Figure 2). The protection and restoration of the main old factory buildings, round kilns, tunnel kilns, and other facilities have been completed, and museums, shops, hotels, creative restaurants, and other facilities have been established; furthermore, a relatively complete ceramic art industry chain is formed (Figure 3). At present, this park not only provides a variety of commercial values as an emerging industrial park, but it is also important for local cultural popularization, art publicity, leisure, and tourism and carries the important social value.

## 2. Research Method for Cultural Landscape Evaluation in Tao Sichuan

### 2.1. Methods of Evaluation

To ensure environmental justice in the construction of the Tao Sichuan landscape and realize the sustainable development of the park, it is necessary to understand the opinions and expectations of different stakeholders. The gathered expectations and opinions on the park must be multidimensional and multilevel. Therefore, in this study, the AHP was chosen to collect opinions. AHP is a multicriteria decision analysis method that is very suitable for this research topic. Indicator selection follows a two-step process: First, the selection of indicators is based on the development status of industrial heritage parks, relevant literature [23] on the sustainable development of cultural tourism, and previous research results. Second, existing problems of the park (e.g., the lack of cultural mining and the imperfection of the comprehensive cultural consumption infrastructure) are summarized, and an evaluation index system is constructed according to the comprehensive, practical, and scientific principles reflected by the index. Based on the realities of the ecological environment and resource exploitation and combined with the current economic and social development, the evaluation criterion layer of the sustainable development of the cultural landscape in Tao Sichuan is constructed. Combined with the problems faced by the landscape in four parts, the index layer, including 12 specific indicators, is constructed.

First, based on the evaluation principle, this article examines the evaluation of the industrial heritage cultural landscape in Tao Sichuan and provides certain suggestions for its transformation through expert opinion matrix analysis and mass culture landscape feeling intensity. The analytic hierarchy process was combined to build the “cultural landscape value assessment level,” including the target layer, standard layer, indicator layer, and content resolution. In this process, the target layer is the public awareness of the value of industrial heritage perceived through the cultural landscape; the standard layer is the specific industrial heritage value of the cultural landscape; the indicator layer is the specific embodiment of each heritage value subset; and content resolution is a detailed explanation of the index layer [24, 25] (Table 1).

Second, the Tao Sichuan industrial heritage landscape evaluation group was probed to identify respondents from both the general public and experts (including representatives from Jiangxi Province Ceramic Industry Corporation, Jingdezhen City Planning Bureau, other experts and scholars, landscape park designers, and construction units). The researcher assessed the public awareness of the ceramic industry through a large number of historical maps and pictures, as well as promotional films and postcards from various periods of Jingdezhen. Community residents from different administrative areas of Jingdezhen city were invited to select their favorite among 86 preprepared photographs of the landscape of Tao Sichuan using a tick-box format. In addition, an electronic questionnaire invited the majority of residents to upload pictures of the Tao Sichuan landscape they took or particularly liked. Because the public's feeling of photographs is incomparable to that of experts, their landscape perception is more authentic.

Third, according to studies on industrial heritage evaluation [26, 27], the industrial heritage landscape is commonly evaluated through aspects of the historical value, artistic value, technological characteristics, social value, and economic value. Therefore, these five aspects were chosen as main evaluation indicators, and sublayers were defined with the help of experts (Table 1).

Fourth, based on the public's selection of photographs of the Tao Sichuan cultural landscape, the distribution of key viewing areas in the park could be obtained and important nodes of the Tao Sichuan industrial heritage cultural landscape could be derived. In addition, design suggestions for the Tao Sichuan cultural landscape were obtained by comparing expert evaluations with public perception.

### 2.2. Research Methodology

The utilized research method mainly includes two value evaluation methods and questionnaire survey methods. The content is divided into two parts: the evaluation of the value of the Tao Sichuan landscape to its industrial heritage by expert groups and the feeling intensity of the public toward the Tao Sichuan cultural landscape. In relation to the expert group and based on the interviews and the value evaluation hierarchy described above, a judgment matrix was constructed by pairwise comparison (Table 2). Each element of each row in A was multiplied and squared *m* times to obtain the vector *W*^∗^ = (*w*1^∗^, *w*2^∗^,…, *w*m^∗^)^T^, where $w_{i}^{*}=\sqrt[{m}]{\prod_{j=1}^{m}a_{ij}}$; then, W^∗^ is normalized to obtain the weight vector *W* = (*w*1^∗^, *w*2^∗^,…, *w*m^∗^)^T^, where *w*_*i*_=*w*_*i*_^*∗*^/∑_*i*=1_^*m*^*w*_*i*_^*∗*^; then, each column element in A is added to obtain the vector, where *s*_*j*_=∑_*i*=1_^*m*^*a*_*ij*_; finally, the factor weight coefficients for each indicator layer and standard layer are obtained.

To ensure that the respondents clearly understand the content of the survey and express their feelings accurately, representative photos were selected as reference objects to evaluate the sensory intensity in the questionnaire survey (see Table 3). A total of 500 questionnaires were sent out, and 478 valid questionnaires were recovered. Among these, 350 were distributed online, of which 326 were valid. Respondents rated the perception of each factor according to their own understanding and then made a subjective judgment based on their own knowledge and experience. Each weighting factor was scored from 1 to 7 (i.e., from weak to strong, respectively), where the scores had to be integers. According to the formula (∑*n*.*e*/*N*) (where *n* = questionnaire number, *e* = feeling degree, and *N* = total number of questionnaires), the standard layer average was obtained. Then, the standard layer average was divided by 3 to obtain the index layer average.

## 3. Research Results and Discussion

### 3.1. Research Results

Through data calculation, the following weights of expert groups on the standard layer of industrial heritage of Tao Sichuan landscape were obtained: A1 (0.2138), A2 (0.4729), A3 (0.0498), A4 (0.0498), and A5 (0.2138). Value index layer weight coefficients are A11 (0.1549), A12 (0.6719), and A13 (0.1732); A21 (0.6370), A22 (0.1047), and A23 (0.2583); A31 (0.1047), A32 (0.2583), and A33 (0.6370); A41 (0.1047), A42 (0.2583), and A43 (0.6370); and A51 (0.1667) and A52 (0.8333). Experts affirmed the artistic value of the industrial heritage park as expressed in the landscape. They identified the scarce historical resources of the ceramic industry, important historical events and figures, factory buildings (clusters), building techniques, placed signs, and intrinsic properties in Tao Sichuan park as important reflections of the industrial heritage in the landscape. The public's perception of the standard layer of Tao Sichuan's industrial heritage cultural landscape is shown in Table 4. The average values of the standard layer of the industrial heritage value of the Tao Sichuan landscape are also shown in Table 4. Among them, the public tends to focus on the ability of the landscape to express both social and artistic values of the industrial heritage, whether the landscape of the park reflects the image of the city, the ceramic culture, the transformation of the park, as well as the artistic feeling and the architectural style of the park.

### 3.2. Discussion of Results

**Figure 4:**
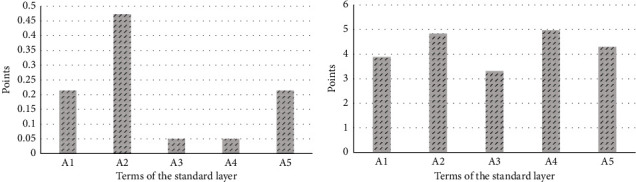
Opinions of experts and the public about the industrial heritage of Tao Sichuan. (a) Weights of experts on the standard layer of the industrial heritage of Tao Sichuan. (b) The public's evaluation of the standard layer of the industrial heritage value of Tao Sichuan.

1. Experts prefer the representation of the relatively stable value of cultural landscapes for industrial heritage. According to the data results, experts believe that the cultural landscape reflects the artistic value of heritage the most. The weight results of each index layer indicate that the value of the circular economy received the highest weight, followed by the historical resources of the scarce ceramic industry, factory buildings (groups), and construction technology. This shows that the expert group focuses on the landscape to promote the development of the circular economy value of the park. This group also focuses on the performance of the relatively stable heritage values of the ceramic industry history, factory buildings, and construction technology, which form an important basis for the landscape transformation of the park.
2. The public's preference for cultural landscape reflects the value of industrial heritage multiplicity. According to the average of public perception at the standard level, the public believes the social value to be the highest intensity of cultural landscape perception. The results of the average feeling of each index layer show that the social life relationship received the highest average, followed by culture, the value of the circular economy, and the art planning of the factory. This result indicates that the public pays attention to the landscape to satisfy and enhance the relationship between social life as the goal. The cultural influence, recycling economy, and plant art planning express multiple heritage values. Although these values will change with the continuous transformation of the object, this result highlights the different landscape needs between the public and experts (Figure 4).

It can be concluded that the expert group and the public group are consistent in their reflections on the heritage value of cultural landscapes. However, more often than not, differences in perception between experts and the general public may lead to a mismatch of specific perceptions in cultural landscapes. For example, experts and the public have completely opposite perceptions of the evaluation of historical and social values embodied in cultural landscapes. Although groups (expert groups) participating in the transformation of industrial heritage ensure the transmission of physical structures to future generations, their efforts are in conflict with the value perception of the general public. Different groups have different experiences with the value of industrial heritage, and therefore, based on landscape justice, the cultural landscape should not only reflect the value of heritage in a balanced way but also the quantitative index of its evaluation and feelings as a necessary guarantee to guide practice (Tables5–10).

## 4. Principles for Enhancing the Cultural Landscape Design of Tao Sichuan Industrial Heritage Park

### 4.1. Increasing the Reflection of the Park Landscape to the Technical Value

As both historical and technical values in industrial heritage are relatively stable, they provide an important basis for the upgrading of cultural landscapes. However, among experts and the general public, the landscape has the lowest perception of technical value, which needs to be improved.

In the light industry, it is difficult for the cultural landscape to independently reflect the scientific nature of industrial technology equipment, industrial science, and technology. Landscape structures can be formed by increasing the sense of industrial technology in the overall landscape space of the factory or by using relevant technologies. An example is the use of industrial waste equipment for the landscape design, which reflects the combination of art and technology, and landscape extension forms in combination with buildings (clusters).

### 4.2. Increasing the Historical Embodiment of Nonarchitectural Elements of the Landscape

Landscape architects perceive sites as historical evidence first, and as aesthetic installations second [28]. There are two reasons why experts apply a high weight for evaluating the historical value of the cultural landscape response. The first reason is that experts possess a relatively good understanding of the history of Tao Sichuan. The second reason is that the industrial historical resources of the historical value, namely, the unique workshop, kiln equipment, production rails, transportation equipment, and other industrial historical resources with construction as the carrier, are relatively intact. However, other public space landscapes not related to the construction reflect little historical value. Therefore, monuments or sculptures with historically representative figures (groups) and event landscape walls can be set up in the open or in semiopen spaces. Then, the landscape of the historical value of the park can expand the public's comprehensive historical experience of industrial heritage.

### 4.3. Expansion of the Landscape Space That Is Conducive for Mass Communication

From the perspective of daily life, the public space system is updated through the continuous updating of public space landscape selection points [29]. Tao Sichuan is a shared resource of the public value. Evaluation data and perception averages should be consulted to identify, for example, which heritage values have not yet been presented and protected in the landscape and which population perceptions or community need to focus on. At present, the landscape of Tao Sichuan is mainly concentrated in consumption areas that have supporting functions, such as restaurants and hotels, while the landscape shaping of public leisure activity areas is missing. In addition, the unique weekly creative market of Tao Sichuan is equally important for the public's understanding of ceramic culture and the communication between shop owners. Therefore, the allocation design of the cultural landscape of Tao Sichuan should start by reflecting public value resources. The landscape design of areas where the public frequently stays should be enhanced, such as creative markets, squares, and roads. The construction of the cultural landscape should be improved in areas where the public easily forms a sense of place and engages in collective communication in daily life.

## 5. Conclusion

Establishing a region's uniqueness mainly depends on the provision of a sense of being local, which is of great significance for urban development. The longer a city has a dominant industry, the more the city becomes a symbol for the entire industry. Tao Sichuan Industrial Heritage Park has witnessed the industrial development of China in general and the development of Jingdezhen city in particular. In this article, AHP is used to construct an evaluation index system, which helps to evaluate the value cognitions of industrial heritage by both experts and the public. Ultimately, their views on the sustainable development of landscape and landscape justice can be reflected. The results showed that experts do not fully agree with the public on the values of industrial heritage. Given this difference, this article proposes design strategies for landscape reconstruction in parks to achieve social justice in the future landscape design. This study is a demonstration of the application of AHP in landscape design research and provides a case study for pursuing landscape justice in urban landscape development. The main limitation of this research is that the survey only focused on local citizens who are familiar with Tao Sichuan. Future research should survey more people from Jingdezhen city, which would increase the reliability of the results.

## Figures and Tables

**Table 1:** Part of the Tao Sichuan cultural landscape value evaluation level.

Target layer	Standard layer	Indicator layer	Content	Photographs of representative cultural landscape in Tao Sichuan
Public awareness of the value of industrial heritage through cultural landscapes	Historical value (A1)	Birth and witness of the industrial history of the Universe Porcelain Factory (A11)	Establishment of industrial enterprises, environment, historical evolution, and development (A11)	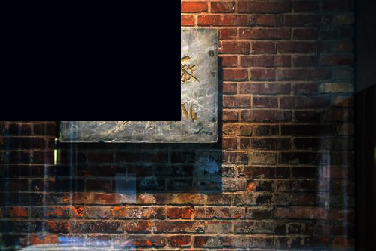 Symbol of old ceramic factory in Tao Sichuan park
		Historical resources and important historical events and figures in the rare ceramic industry (A12)	Age, type, and unique particularity, rarity, historical events, and representative figures (A12)	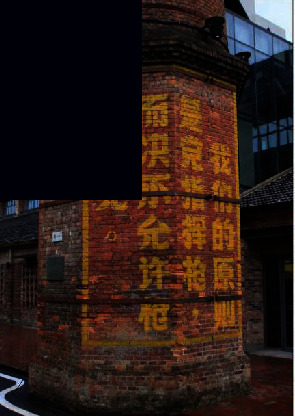 The original factory production slogan of Tao Sichuan park
		Authenticity and integrity of relevant historical information (A13)	Environmental, time, space, group, building (main body of the plant, material, decoration, and other historical information (A13)	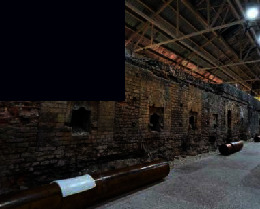 Original site of firing equipment for ceramic production

Public awareness of the value of industrial heritage through cultural landscapes	Art value (A2)	Plant buildings and buildings (A21)	Bauhaus style, serrated and herringbone barns, steel construction, red brick material, decoration, colors (A21)	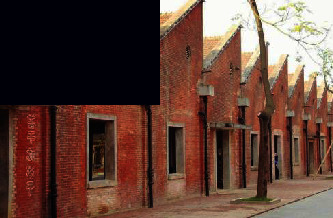 Production workshop
		Factory art planning (A22)	Overall campus planning, spatial structure of the plant, technical production structure, public environment, renovation of facilities, and division of functional areas (A22)	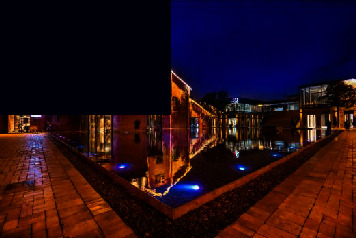 Renovated park square
		Artistic expression of the structure of industrial facilities (A23)	Public artworks related to industrial materials, equipment, and themes (A23)	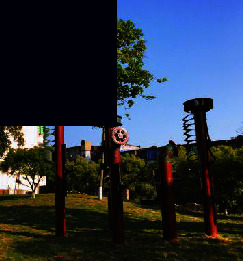 Public art (the material is derived from parts of the production equipment)

Public awareness of the value of industrial heritage through cultural landscapes	Technical value (A3)	Impact of industrial technology (A31)	Production arts, and product technology (A31)	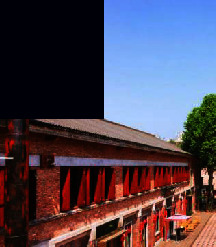 Hydrothermal circulation system and the smoke exhaust pipe of the ceramic production workshop of Tao Sichuan park
		Scientific and technical nature of industrial facilities and equipment (A32)	Industry advanced equipment (A32)	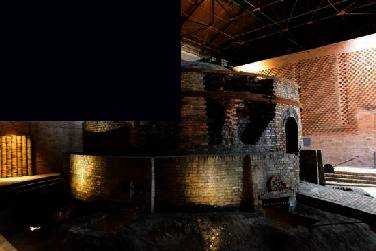 Ceramic production equipment: steamed bun kiln
		Architectural technology (A33)	Structure, material, and construction (A33)	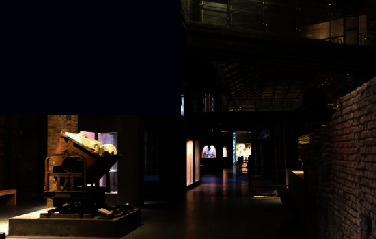 Spatial structure for firing and transporting products in production workshops

Public awareness of the value of industrial heritage through cultural landscapes	Social value (A4)	Relationship of social life (A41)	Connection between industrial heritage and social life, ideas, and customs (A41)	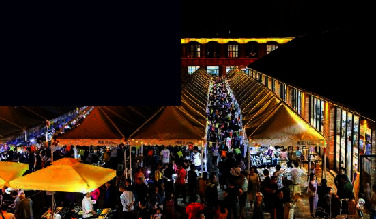 Ceramic products trading activities in the market of Tao Sichuan park
		Impact of culture (A42)	Educational, social, industrial cultural concepts (A42)	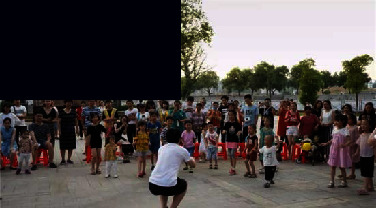 Children's outdoor education programme at Tao Sichuan campus
		Placement of identity and intrinsic properties (A43)	Logo, identity, sense of belonging, recognition, historical memory, inheritance of industrial spirit (A43)	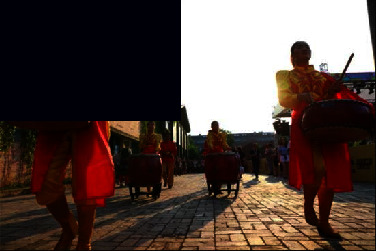 A scene from the kiln firing ceremony at the park

Public awareness of the value of industrial heritage through cultural landscapes	Economy value (A5)	Intrinsic economic value (A51)	Local shops, activities, fairs, restaurants, education, exhibition, and charity activities (A51)	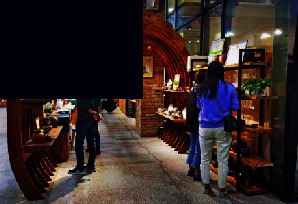 Local shops
		Recyclable economic value (A52)	Cooperation with units outside of commercial activities (A52)	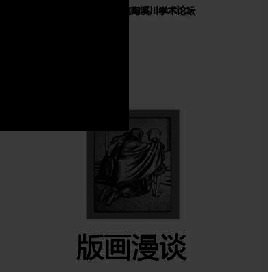 Cooperation with Beijing University

**Table 2:** Pairwise comparison.

	Comparison between any two means
1	Factor *i* is as important as factor *j*
3	Factor *i* is slightly more important than factor *j*
5	Factor *i* is significantly more important than factor *j*
7	Factor *i* is very important compared with factor *j*

**Table 3:** Questionnaire statistics on the perception of the public regarding the landscape of the Tao Sichuan Industrial Heritage Park.

Indicator layers	Criterion layer corresponding to the problem number	Degree of feeling (quantitative statistics)						
		1	2	3	4	5	6	7
Historical value B1	Question 1	14	24	87	115	53	21	12
	Question 2	9	17	68	100	98	20	14
	Question 3	10	30	102	125	40	10	9

Art value B2	Question 4	5	4	25	82	126	59	25
	Question 5	2	4	18	93	140	40	29
	Question 6	4	3	16	116	100	56	31

Technical value B3	Question 7	36	32	96	80	37	21	24
	Question 8	61	29	95	72	37	15	17
	Question 9	74	43	112	51	23	9	14

Social value B4	Question 10	3	4	11	58	107	57	86
	Question 11	7	3	27	80	98	52	59
	Question 12	9	11	36	121	77	34	38

Economic value B5	Question 13	9	9	36	101	82	51	38
	Question 14	2	1	11	110	109	60	33
	Question 15 (16)	9 3	22 6	55 21	130 69	52 44	13 19	18 17

**Table 4:** The degree of public perception of the industrial heritage landscape of Tao Sichuan.

Standard layers (mean)	Index layer corresponding number	Mean value of the index layer
Historical value A1 (3.8875)	Question 1 (“Does the park convey a sense of history?”)	3.8589
	Question 2 (“What is the degree of integrity of preservation of building structures, machinery, equipment, and production materials?”)	4.1564
	Question 3 (“Is there much information retained by history?”)	3.6779

Artistic value A2 (4.8354)	Question 4 (“Is the architectural style outstanding?”)	4.8313
	Question 5 (“What is the artistic feeling of the overall park?”)	4.8436
	Question 6 (“Is the outdoor landscape sketch satisfactory?”)	4.8313

Technical value A3 (3.3129)	Question 7 (“Are you familiar with the ceramic production line or ceramic production process?”)	3.6411
	Question 8 (“Are you familiar with the production function of Tao Sichuan?”)	3.3313
	Question 9 (“Do you understand the construction technology of existing building workshops?”)	2.9663

Social value A4 (4.9714)	Question 10 (“Does the landscape in the park improves the image of Jingdezhen?)	5.3834
	Question 11 (“Does landscape spread ceramic culture?”)	4.9970
	Question 12 (“Does the landscape in the park carries an industrial spirit?”)	4.5337

Economic value A5 (4.2969)	Question 13 (“Is the park transportation convenient?”)	4.6656
	Question 14 (“Does Tao Sichuan's current transition succeed?”)	4.9601
	Question 15 (16) (“What is your desire to spend? Are employment opportunities provided?”)	3.2654

**Table 5:** Evaluation matrix of the historical value (A1) in the cultural landscape of Tao Sichuan Industrial Heritage Park.

	A11	A12	A13
A11	1	1/7	1/3
A12	7	1	5
A13	3	1/5	1

**Table 6:** Evaluation matrix of the artistic value (A2) in the cultural landscape of Tao Sichuan Industrial Heritage Park.

	A21	A22	A23
A21	1	5	3
A22	1/5	1	1/3
A23	1/3	3	1

**Table 7:** Evaluation matrix of the technology value (A3) in the cultural landscape of Tao Sichuan Industrial Heritage Park.

	A31	A32	A33
A31	1	1/3	1/5
A32	3	1	1/3
A33	5	3	1

**Table 8:** Evaluation matrix of the social value (A4) in the cultural landscape of Tao Sichuan Industrial Heritage Park.

	A41	A42	A43
A41	1	1/3	1/5
A42	3	1	1/3
A43	5	3	1

**Table 9:** Evaluation matrix of the economic value (A5) in the cultural landscape of Tao Sichuan Industrial Heritage Park.

	A51	A52
A51	1	1/5
A52	5	1

**Table 10:** Judgment matrix of the cultural landscape value of Tao Sichuan Industrial Heritage Park.

A	A1	A2	A3	A4	A5
A1	1	1/3	5	5	1
A2	3	1	7	7	3
A3	1/5	1/7	1	1	5
A4	1/5	1/7	1	1	1/5
A5	1	1/3	5	5	1

## Data Availability

The authors confirm that the data supporting the findings of this study are available within the article and its supplementary materials.
